# In Vitro Chemopreventive Properties of Green Tea, Rooibos and Honeybush Extracts in Skin Cells

**DOI:** 10.3390/molecules21121622

**Published:** 2016-11-25

**Authors:** Tandeka U. Magcwebeba, Pieter Swart, Sonja Swanevelder, Elizabeth Joubert, Wentzel C. A. Gelderblom

**Affiliations:** 1 Department of Biochemistry, Stellenbosch University, Private Bag X1, Matieland (Stellenbosch) 7602, South Africa; tmagcwebeba@sun.ac.za (T.U.M.); pswart@sun.ac.za (P.S.); 2 Biostatistics Unit, South African Medical Research Council, P.O. Box 19070, Tygerberg 7505, South Africa; sonja.swanevelder@mrc.ac.za; 3 Post-Harvest and Wine Technology Division, Agricultural Research Council (Infruitec-Nietvoorbij), Private Bag X5026, Stellenbosch 7599, South Africa; joubertL@arc.agric.za; 4 Department of Food Science, Stellenbosch University, Private Bag X1, Matieland (Stellenbosch) 7602, South Africa; 5 Institute of Biomedical and Microbial Biotechnology, Cape Peninsula University of Technology, P.O. Box 1906, Bellville 7535, South Africa

**Keywords:** anti proliferative, pro-apoptotic, flavanols, herbal teas, skin cancer

## Abstract

The chemopreventive properties of the herbal teas rooibos (*Aspalathus linearis*) and honeybush (*Cyclopia* spp.) have been demonstrated on mouse skin in vivo but the underlying mechanisms are not clear. The aim of the current study was to determine the anti-proliferative and pro-apoptotic activity of methanol and aqueous extracts of rooibos and two *Cyclopia* species in different skin cells, using green tea (*Camellia sinensis*) as a benchmark. Extracts were also characterised for their major individual polyphenols by high performance liquid chromatography and spectroscopically for the total polyphenol (TP) groups. The methanol extract of rooibos, containing higher levels of polyphenols than its aqueous extract, displayed similar activity to green tea as it selectively targeted premalignant cells by inhibiting cell proliferation at lower concentrations whilst inducing apoptosis via membrane depolarisation at higher concentrations. Specific roles of the major rooibos dihydrochalcones and flavanol/proanthocyanidin-type (FLAVA) compounds are likely to be involved. The aqueous extracts of the *Cyclopia* species were more active against cell proliferation and at inducing apoptosis which was associated with a higher FLAVA content and a reduced TP/FLAVA ratio. In contrast, their methanol extracts exhibited a cytoprotective effect against apoptosis which was related to their monomeric xanthone and flavanone content. The underlying chemopreventive properties of green tea and the herbal teas appear to be associated with diverse and complex monomeric/polymeric polyphenolic cell interactions.

## 1. Introduction

The incidence of skin cancer, resulting from UVB irradiation, has become a global concern as it continues to rise rapidly with non-melanoma skin cancers, comprising of basal cell carcinoma and squamous cell carcinoma, being the most frequently diagnosed in Caucasian populations [1]. It is currently estimated that 1 out of 3 cancers diagnosed is skin cancer, with an average of 2 to 3 million cases of non-melanoma and 132,000 melanoma being reported annually [2]. Existing prevention strategies and treatment options have limitations which contribute to the morbidity and mortality rate of this disease. These include low-compliance to sun avoidance behavioral changes as well as costly and highly invasive treatment modalities which have a high recurrence rate [1]. The development of non-melanoma skin cancers, like any other cancer, progresses through three distinct stages, namely initiation, promotion and progression [3,4]. One of the hallmarks of cancer promotion, caused by chronic exposure to UVB-irradiation and other known tumor promoters, is the induction of cellular hyperproliferation. This hyperproliferative state overcomes the latency of initiated cells and leads to hyperplastic transformation during cancer development [5,6]. Activation of cell survival mechanisms and dysregulation of apoptosis, caused by an impairment in death receptor signalling and mutations in the p53 gene, are key determinants regulating this process in damaged cells [7,8]. The apoptotic-resistance conferred by these events allows for the clonal expansion of transformed cells to progress into malignant skin tumours. Consequently, induction of apoptosis to eliminate transformed cells has been identified as an important intervention strategy in the prevention of carcinogenesis [4].

Some dietary components have been shown to have the ability to interfere with the process of cancer development and one of the mechanisms that has been suggested to play an important role in their chemopreventive properties is the removal of damaged/mutated via apoptosis [9,10]. The chemopreventive properties of dietary products such as fruits, vegetables and tea have been associated with their high polyphenolic content [11,12]. Consequently, much of the research focus has been on the development of polyphenol-enriched natural products to be used as chemopreventive agents. Green tea, produced from the unfermented leaves of *Camellia sinensis*, is among the most extensively studied natural dietary agents in skin and its biological activity against carcinogenesis has mainly been attributed to the presence of flavanols, the catechins, with epigallocatechin gallate (EGCG), as one of the most studied constituents [13,14]. Numerous in vivo studies have indicated that green tea and its flavanols can protect against carcinogenesis in skin by reducing tumor incidence, multiplicity, growth and malignant conversion [15,16,17,18]. These chemoprotective effects can be achieved either through oral administration or topical application [19,20,21] with the latter reported as a more effective route of application. Studies in cell cultures and animal models have proposed various mechanisms for the anti-carcinogenic activity of green tea and its flavanols in skin [14]. These include reduction of cell proliferation via cell cycle arrest and induction of pro-apoptotic events that mainly involve membrane depolarisation, caspase activation, cytochrome c release and DNA fragmentation via PARP cleavage [22,23,24].

The anti-cancer properties of the South African herbal teas, rooibos (*Aspalathus linearis*) and honeybush (*Cyclopia* spp.), demonstrated in rat liver, oesophagus, as well as mouse skin carcinogenesis models, have also been attributed to their polyphenolic compounds [25,26,27,28]. The protective effects of two of the major honeybush polyphenols, the xanthone mangiferin and the flavanone hesperidin, have been reported in a pre-exposure UVB-induced skin carcinogenesis model and the proposed mechanisms were associated with the modulation of oxidative stress and inhibition of cell proliferation [28]. In a more recent study, extracts of rooibos and honeybush reduced the viability of different skin cells by inhibiting the production of ATP and this was closely related to high levels of monomeric polyphenols and flavanol/proanthocyanidin-type (FLAVA) compounds [29]. However, since a reduction of ATP production in cells was effected, a specific role for the herbal tea extracts in the induction of cell cycle arrest and apoptosis via mitochondrial dysfunction was suggested. This hypothesis was further strengthened by the anti-proliferative and pro-apoptotic activity exhibited by these herbal tea extracts in UVB-exposed HaCaT skin keratinocytes [30]. Therefore, the present study is a continuation to gain more insight into the effect of the same rooibos and honeybush extracts on cell proliferation and apoptosis in different skin cell culture systems. The effects were related to their polyphenolic constituents using green tea as benchmark.

## 2. Results

### 2.1. Effect of Green Tea and Herbal Tea Extracts on Cell Proliferation

Green tea and rooibos extracts exhibited the highest activity against the proliferation of different skin cells with the methanol extracts being significantly (*p* < 0.05) more effective than the aqueous extracts (Table 1). Both green tea and rooibos extracts inhibited the proliferation of premalignant cells (HaCaT) and cancer cells (CRL 7762) at significantly (*p* < 0.05) lower concentrations than the normal cells (CRL 7761) with the rooibos methanol extract displaying the highest activity against the cancer cell line. The methanol extract of green tea exhibited similar activity in the premalignant and cancer cell lines whilst its aqueous extract was more active against premalignant cells. Contrary to green tea and rooibos extracts, the aqueous extracts of honeybush, except for *C. subternata* against premalignant cells, exhibited a significant (*p* < 0.05) higher activity than their methanol extracts (Table 1). The aqueous extracts of the two *Cyclopia* spp. inhibited the proliferation of normal cells at concentration lower than those required for premalignant and the cancer cells. The activity of the methanol extracts differed, with the *C. subternata* extract exhibiting a similar activity against all three cell lines, whilst *C. intermedia* targeted normal and cancer cells at similar concentrations.

### 2.2. Induction of Pro-Apoptotic Caspase-3 Activity

The methanol extracts of green tea and rooibos extracts induced caspase-3 activity in a dose-dependent manner in the different skin cell lines with the cancer cells being more resistant (Table 2). Depending on the dose, the methanol extract of green tea exhibited a higher pro-apoptotic activity when compared to its aqueous extract in the premalignant and normal cells while no difference was noticed in the cancer cells, even at a higher extract concentration. The methanol and aqueous rooibos extracts tended to effect similar pro-apoptotic effect against the skin cells at the different concentrations. The premalignant cells were the most sensitive cell line, while cancer cells exhibited the weakest response for both green tea and rooibos extracts. The induction of apoptosis by both extracts of green tea and rooibos was closely related to the reduction of cell viability as an inverse relationship existed between induction of caspase-3 activity and the ATP content.

In contrast, the aqueous extracts of both *Cyclopia* species induced a dose-dependent caspase-3 activity in the skin cells although to a far lesser extent compared to green tea and rooibos extracts, evident from the higher concentrations required (Table 3). The methanol extract of *C. intermedia* lacked any significant effects on the induction of caspase-3 activity in the different cell lines and even reduced the effect in the cancer cells. No significant effect on caspase-3 activity by the methanol extract of *C. subternata* was noticed in the normal and cancer cells. A significant increase was, however, noticed in the premalignant cells at the highest concentration. The premalignant cells were the most sensitive cell line with the normal and cancer cells being more resistant showing only a slight increase in activity for the aqueous extracts above the solvent control treatment. As described above for green tea and rooibos extracts, the induction of caspase-3 activity by the aqueous extracts of the *Cyclopia* species was closely associated with the reduction in cell viability. 

#### 2.2.1. Relationship between Pro-Apoptotic Effect and Cell Viability

When considering the relationship between caspase-3 fold increase and cell viability, the methanol extracts of both green tea and rooibos exhibited a strong negative correlation (Table 4). A similar response was noticed for the aqueous extracts of green tea in premalignant, cancer and normal cells. The aqueous extract of rooibos displayed a similar effect, except for the moderate negative correlation in normal cells. On the other hand, the aqueous extracts of *C. intermedia* and *C. subternata* exhibited a strong negative relationship in premalignant and normal cells, but a moderate relationship was observed in cancer cells (Table 4). The methanol extracts of the honeybush extracts exhibited differential effects where the reduction in cell viability was associated either with an increase in caspase-3 for premalignant cells or decrease/lack of effect in cancer cells. Consequently the methanol extract of *C. subternata* showed a strong negative correlation in premalignant cells, a weak negative correlation in normal cells whilst no correlation was observed in cancer cells. In contrast, the methanol extract of *C. intermedia* exhibited a moderate negative and positive correlation in the premalignant cells and cancer cells, respectively lacking any correlation in normal cells.

#### 2.2.2. Morphological Alteration in Normal Cells Associated with Induction of Apoptosis

**Figure 1:**
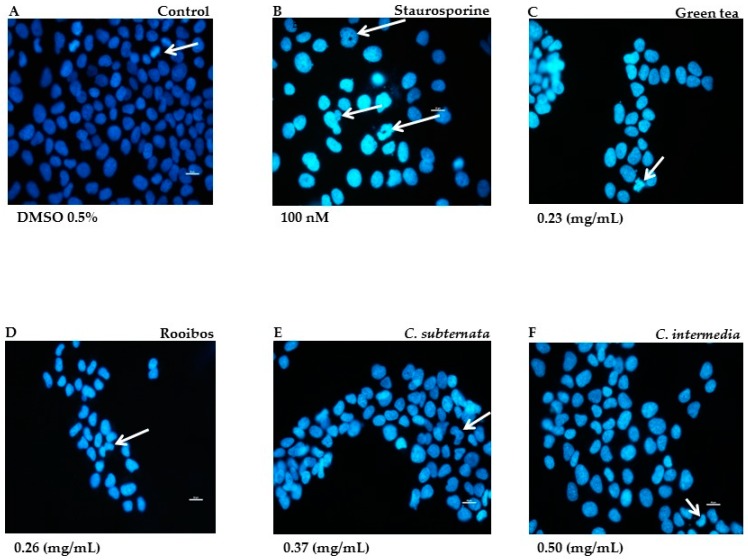
Morphological features in cells stained with Hoechst after 24 h incubation in the presence of green tea and the various tea/herbal tea extracts. Untreated cells displayed dimly stained nuclei; arrow indicates pro-metaphase (**A**). Positive control cells were treated with staurosporine (100 nM; (**B**)) Arrows indicate unstained nucleolar region (chromatin condensation) nuclear fragmentation and formation of apoptotic bodies. Cells exposed to methanol extract of green tea (**C**) show nuclear fragmentation. Rooibos (**D**) treated cells show crescent shape figures (arrow). Effects of aqueous extracts *C. subternata* and *C. intermedia* depicted in (**E**,**F**) also displayed similar features, but also with blebbing. Bar = 20 µM; Magnification 40×.

The Hoechst nuclear DNA stain provided qualitative data on the mode and degree of apoptotic cell death induced by the different tea and herbal tea extracts as demonstrated in the normal cell line. In the untreated control cells exhibited a low intensity blue colour and reflected cell proliferation as compact DNA fluoresced brightly, indicating pro-metaphase during mitosis (Figure 1A). Positive control cells treated with staurosporine were brightly stained; arrow heads indicate unstained nucleolar region associated with chromatin condensation, nuclear fragmentation and the formation of apoptotic bodies (Figure 1B). Cells exposed to the methanol extract of green tea displayed smaller nuclei, were fewer in field, brightly stained and exhibited nuclear fragmentation (Figure 1C). Rooibos also showed similar features but instead of nuclear fragmentation, cells exhibited crescent-shaped structures associated with chromatin condensation (indicated with arrows in Figure 1D). The aqueous extract of *Cyclopia subternata* also exhibited crescent shape structures in addition to nuclear blebbing (arrow in Figure 1E). Similar features were also noticed following exposure of the cells to the aqueous extract of *C. intermedia* (Figure 1F).

#### 2.2.3. Characterisation of Rooibos-Induced Apoptosis in Normal Cells by Flow Cytometry

**Figure 2:**
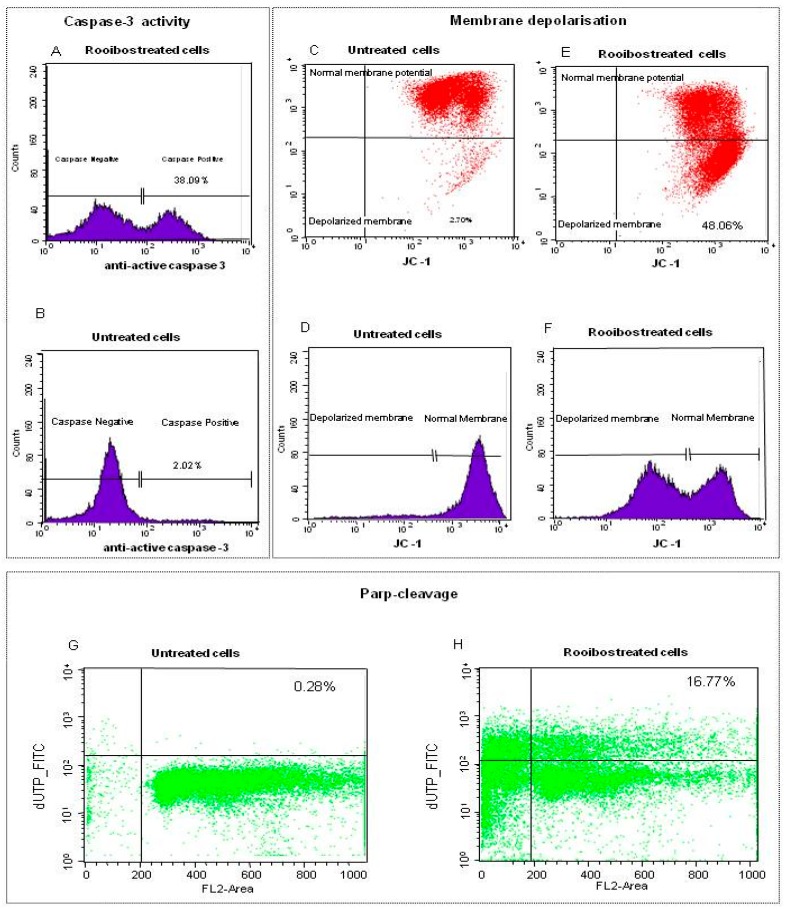
Flow cytometric analysis of normal cells treated with rooibos methanol extract (0.32 mg/mL). Caspase-3 activity was induced in treated cells (**A**) when compared to untreated control cells (**B**). Untreated cells also exhibited an intact polarized membrane as red fluorescent JC-1 aggregates accumulated in the mitochondria of the untreated viable cells (**C**,**D**), while treatment caused membrane depolarisation with JC-1 leaking into the cytoplasm (**E**,**F**). High concentration of non-apoptotic cells in the lower channel indicated very little DNA fragmentation in control cells (**G**). Treated cells exhibited DNA fragmentation as damaged cells were located in the upper panel while most of the cells were pro-apoptotic (sub G1; to the left of the vertical axes; (**H**)). Cell towards the left of the vertical line are in sub-G1 or pro-apoptotic while most of the non-apoptotic cells were in G0/G1 (between 200 to 400 FL2 Area).

The methanol extract of rooibos exhibited the highest apoptotic activity against premalignant and normal cells when considering only the herbal teas. Since the focus of this study was to determine if reduction of ATP and induction of apoptosis were associated with mitochondrial dysfunction in non-mutated cells with normal differentiation, further analysis of the rooibos methanol extract was conducted in normal cells utilising flow cytometry. Upon treatment of the cells with the methanol extract at a concentration equaling the IC_50_ value for reduction of ATP content, more than 30% of the treated cells displayed caspase-3 activity compared to the untreated cells (Figure 2A,B). When monitoring the uptake of JC-1 into the mitochondria the red fluorescent JC-1 aggregates accumulated in the mitochondria of untreated cells, with only a small population of cells exhibiting a reduction in fluorescence, thus indicating intact polarised mitochondrial membrane (Figure 2C,D). In contrast, treatment with the rooibos methanol extract disrupted mitochondrial potential as a significant amount of JC-1 leaked into the cytoplasm, indicated by an intense fluorescence in the FL-2 channel (second lower quadrant), as well as a shift to the left in the histogram (Figure 2E,F). Untreated cells did not show any increase in DNA fragmentation (PARP cleavage), whilst the treated cells exhibited DNA fragmentation associated with the late stages of apoptosis (Figure 2G,H).

### 2.3. Differences in Polyphenol Content and Specific Ratios between Methanol and Aqueous Extracts

Previously it was shown that the aqueous extracts of green tea and the herbal teas have a lower total phenolic content than their corresponding methanol extracts (Table 5) [28]. Although levels of total polyphenols (TP), flavanol/proanthocyanindin (FLAVA) and total monomeric flavanols, specifically EGCG, was significantly reduced in the aqueous extract of green tea, the TP/FLAVA ratio was similar to that of the methanol extract. The caffeine content of green tea in the aqueous extract (40.1 ± 0.3 µg/mg extract was however, significantly (*p* < 0.05) reduced when compared to the methanol extract (57.5 ± 1.6 µg/mg extract), although not to the same extent as the TP constituents. The methanol extract of rooibos, contained high levels of the dihydrochalcones (DHC) constituting the major components of the total monomeric polyphenolic compounds. The levels of these constituents was significantly reduced in the aqueous extracts. However, rooibos contained relatively low levels of FLAVA which resulted in a markedly higher TP/FLAVA ratio when compared to green tea (Table 5). The monomeric polyphenols of the honeybush extracts, specifically the xanthones, mangiferin and isomangiferin and the flavanone, hesperidin, were significantly decreased in the aqueous extracts (Table 5). Of interest was that the FLAVA content was increased in the aqueous extracts of both honeybush species, resulting in a prominent decreased in the TP/FLAVA ratio when compared to the methanol extract. A higher TP/FLAVA ratio was thus obtained for the methanol extract of the honeybush teas when compared to rooibos and green tea.

## 3. Discussion

As cancer cells are postulated to be more susceptible to oxidative stress, their altered energy metabolism has been identified as a primary target for the preferential killing of damaged/mutated cells during chemoprevention. Subsequently, several in vitro studies indicated the ability of natural and synthetic compounds to selectively target cancer cells by inducing mitochondrial dysfunction which leads to enhanced oxidative stress, cell cycle arrest and apoptosis [31,32,33,34]. Extracts of unfermented plant material of rooibos and honeybush species have been shown to reduce cell viability by decreasing the ATP content in skin cells, suggesting that they may alter the growth of skin cancer cells by disrupting mitochondrial function [29,30]. Since mitochondrial dysfunction is known to suppress the growth of cancer cells through cell cycle arrest and apoptosis [35], the anti-proliferative and pro-apoptotic effects of the herbal tea extracts were of interest. The aim of the current study was to evaluate the modulatory activity of rooibos and honeybush herbal teas against cell proliferation and apoptosis in premalignant, normal and cancer skin cells in order to determine whether cancer cells can be selectively targeted. The different green tea and herbal tea extracts inhibited the proliferation of skin cells at concentrations that were lower than the IC_50_ values for the same extracts previously reported for the reduction in cell viability (ATP production) [29]. This would imply that inhibition of cell proliferation by these extracts does not result from complete cell loss, but may rather be the result of other mechanisms that involve induction of cell cycle arrest at the lowest concentrations which then leads to the induction of apoptosis at higher concentrations. 

Green tea methanol extract selectively inhibited the proliferation of the premalignant and cancer cell lines to a greater extent when compared to the normal cells at the concentrations tested. However, at the highest concentration equaling IC_50_ [29], both the methanol and the aqueous extracts, induced apoptosis with the methanol extract being more effective in premalignant and normal cells, while both extracts exhibited a weak response against cancer cells only at the highest concentrations. Caspase-3 activation by green tea polyphenols is mostly associated with the disruption of the mitochondrial electron transport chain, ROS accumulation and changes in the membrane potential [24,36,37]. Since green tea polyphenols mediates apoptosis via the mitochondrial pathway in skin cancer cells [23,24], it is likely that induction of apoptosis in the current study also results from mitochondrial dysfunction. However, the pro-apoptotic activity of the green tea extracts was not selective for the cancer cells as was noticed for the inhibition of cell proliferation as the former cells exhibited a weaker apoptotic response than normal cells. This could be due to the unique metabolic phenotype of cancer cells involving aerobic glycolysis that is associated with a state of apoptotic resistance [38]. In contrast, the premalignant cells were the most sensitive cell line to the pro-apoptotic activity indicating that the green tea extracts target pre-cancerous skin cells with irreparable damage. This verifies findings on chemopreventive properties involving pro-apoptotic activity of green tea extracts and its major compounds, EGCG and caffeine, against unrepaired DNA damage in cells [39,40]. In this regard the premalignant HaCaT cell line is known to have p53 mutations and hence it is more sensitive to the induction of apoptosis [41,42]. 

The methanol extract of green tea containing higher levels of polyphenols exhibited higher activity than its aqueous extract which implicated the role of flavanols in anti-proliferative and pro-apoptotic effects of the extracts in skin cells. In this regard, the anti-proliferative activity of the major polyphenolic compound, EGCG, has been demonstrated in skin cells. These studies indicated that, at lower concentrations, EGCG selectively inhibits the proliferation of cancer cells by inducing cell cycle arrest at the G_1_ and S-phases. At higher concentrations EGCG and other green tea polyphenols selectively induce apoptosis in cancer cells via the mitochondrial pathway [23,24]. The proposed mechanisms involve modulation of the Bcl-2 family, release of cytochrome *c*, activation of caspase-3-cascade leading to cleavage of PARP and subsequent DNA degradation. Therefore, selective cytostatic and pro-apoptotic activity of green tea extracts against cancer cells and premalignant cells in the current study is likely to proceed via the same mechanisms and may be mediated by flavanols. Although the TP to flavanol/proanthocyanidin (TP/FLAVA) ratios of the aqueous and methanol extracts were similar, the reduced response of the aqueous extract is associated with the lower TP, FLAVA and flavanol content, specifically the EGCG concentration. However, caffeine, which is one of the major constituents of green tea may also contribute to the pro-apoptotic effects of green tea extracts as it is known to promote apoptosis of unrepaired keratinocytes [42]. 

The rooibos extracts displayed similar effect to green tea against cell proliferation and induction of apoptosis in cells. However, both extracts were more effective than green tea extracts at selectively inhibiting the proliferation of cancer cells. The pro-apoptotic activity of rooibos extracts, similar to those of green tea extracts, was more prominent in the premalignant cells, which suggested that they may also preferentially target genetically altered cells. The increased activity exhibited by the rooibos methanol extract contains high levels of TP, FLAVA and DHCs, as well as other monomeric polyphenols, implicated the role of rooibos monomeric flavonoids and polymeric polyphenols anti-proliferative and pro-apoptotic activity of the extracts. As rooibos contained relatively low levels of FLAVA resulting in a higher TP/FLAVA ratio than green tea, indicates the importance of polyphenol diversity of their monomeric compounds in the chemopreventive properties. In this regard, the selective cytotoxicity of rooibos flavonoids, including luteolin, rutin and vitexin have been demonstrated against different cancer cell lines [43,44,45]. Mechanistic studies indicated that most of these compounds induce cell cycle arrest either at G_0_/G_1_ phase, G_2_/M phase or S-phase while apoptosis is mediated via the mitochondrial pathway [45]. An in silico study focusing on the specificity of the anti-cancer activity of natural compounds indicated that aspalathin, the major compound in rooibos, as well as the flavonoids, rutin, isoorientin and isovitexin, inhibit the activity of the Bcl-2 proteins as an underlying mechanism in their pro-apoptotic activity [46]. Flow cytometric analysis utilizing normal skin cells indicated that the rooibos methanol extract of rooibos arrests cell cycle progression of normal cells at the G_0_/G_1_. Therefore, the mechanisms underlying the anti-proliferative activity of rooibos extracts in the different skin cells are likely to involve the delay of cell cycle progression at G_0_/G_1_ while apoptosis is mediated via the mitochondrial pathway. The latter is evident from flow cytometric analyses indicating the induction of caspase-3 activity and membrane depolarisation (JC-1), as well as DNA fragmentation. These events are associated with mitochondrial dysfunction and late stages of apoptosis [47,48]. Since membrane depolarisation and apoptosis were effected at a concentration equaling the IC_50_ concentration for ATP inhibition [29], it provided evidence that reduction of cell viability results in mitochondrial dysfunction that leads to cell cycle arrest of cells in the G_1_/G_0_ phase at lower concentrations whilst inducing apoptosis at higher concentrations. Apoptotic activity was confirmed by Hoechst stain which indicated numerous morphological changes associated with early and late stages of apoptosis.

In the previous study, the reduction of skin cell viability induced by rooibos was attributed to both the monomeric rooibos flavonoids and polymeric flavanol/proanthocyanidin constituents [29]. The latter was proposed to play a more important role in the lipid environment while the monomeric flavonoids would likely intercalate in the lipid/aqueous interphase [49]. It would appear that these compounds also play an important role in the reduction of cell proliferation and induction of apoptosis in skin cells. However, specific interactions between reactive constituents seem to play a role in the cytotoxic effects of rooibos in skin cells. This is deduced from the fact that the methanol and aqueous extracts exhibited a comparable TP/FLAVA ratio with very similar effects on cell viability. Therefore, interesting interactive dynamics and specific rooibos TP/FLAVA ratios seem to exist when utilising complex mixtures. The reactive constituents of the methanol extract appear to exhibit synergistic or additive effects while the reduction in the aqueous extract with the presence of more water soluble compounds seem to alter these interactive dynamics. 

The aqueous extracts of honeybush, unlike green tea and rooibos, exhibited anti-proliferative and pro-apoptotic activity in the skin cells than the methanol extracts primarily targeted the premalignant cells. On the other hand, the methanol extracts exhibited a far lower apoptotic effect in the premalignant and normal skin cells while in cancer cells, the methanol extract of *C. intermedia* exhibited a cytoprotective effect by reducing caspase-3 activity at the highest concentrations whilst *C. subternata* had no significant effect. As cancer cells are known to have a pro-oxidative status [50], it is likely that the cytoprotective effect could be related to a reduction of cellular oxidative stress resulting from the antioxidant properties of xanthones and flavanones. In spite of the fact that cell viability was reduced it was not associated with an induction of apoptosis which suggest more complex polyphenol/cell interactions. In this regard, the methanol extract of *C subternata*, containing reduced levels of the xanthones and flavanones, exhibited a lower protective effect against the induction of apoptosis. It was previously suggested that the monomeric polyphenols of honeybush such as mangiferin and hesperidin may confer a protective effect against oxidative stress in skin cells [30]. This was confirmed by the higher cytotoxic effects displayed by the aqueous extracts containing lower levels of these polyphenols presumably due to their lower solubility in the aqueous phase [29]. Since the aqueous extracts contain a higher FLAVA (flavanol/proanthocyanidin) content the anti-proliferative activity and pro-apoptotic activity of honeybush species is likely to be mediated by these constituents. The increased activity against the inhibition of cell proliferation and induction of apoptosis seem to be dependent on a specific TP to FLAVA ratio which, is reduced in the aqueous extracts due to a high FLAVA content. This was also reflected in a recent study where the aqueous extracts of these two honeybush species further enhanced UVB-induced apoptosis in skin keratinocytes which was related to a high FLAVA content [30]. The underlying mechanisms in their anti-proliferative and pro-apoptotic activity, although less active, may involve similar mechanisms to those described for green tea and rooibos. Of interest is that hesperidin has been shown to induce apoptosis by regulating Bcl-2 proteins via interacting with the flavonoid/protein binding site [46]. In this regard, proanthocyanidins from different plant origins have been shown to inhibit the proliferation of different carcinoma cells, thus emphasizing their possible role as chemopreventive agents [51,52].

## 4. Materials and Methods

### 4.1. Reagents and Assay Kits

Heat inactivated fetal bovine serum (FBS) was purchased from Invitrogen (Carlsbad, CA, USA). RPMI-1640, Dulbecco’s modified Eagle’s medium (DMEM), l-glutamine, trypsin-versene and Hank’s buffered salt solution (HBSS) were obtained from Lonza (Braine-l’Alleud, Belgium). Dulbecco’s phosphate-buffered saline (DPBS), dimethyl sulfoxide (DMSO), Hoechst 33342 and staurosporine were purchased from Sigma-Aldrich (St. Louis, MO, USA). The cell proliferation ELISA, BrdU chemiluminescent kit was obtained from Roche (Mannheim, Germany), the CellTiter-Glo^®^ Luminescent cell viability assay kit and the caspase-3/7 assay kit from Promega (Madison, WI, USA) and the BD^TM^ mitoscreen (JC-1) kit (BD Bioscience, San Jose, CA, USA), PE active caspase-3 apoptosis kit and APO-DIRECT™ kit from BD™ Pharmingen (San Diego, CA, USA).

### 4.2. Preparation of Extracts and Polyphenols Analyses

Aqueous and methanol extracts of “unfermented” (unoxidized) rooibos (*Aspalathus linearis*), “unfermented honeybush” (*C. intermedia* and *C. subternata*) and green tea (*Camellia sinensis*), previously prepared (in triplicate) and utilized during in vitro studies on skin cell viability [29] and anti-inflammatory effects in a UVB/Keratinocyte (HaCaT) model [30], were used. Phenolic characterisation of the extracts in terms individual polyphenol content by high performance liquid chromatography-diode array detection and global parameters, i.e., total polyphenol (TP) and flavanol/proanthocyanidin (FLAVA) content, using colorimetric assays, has been described previously [29]. Total polyphenol content refers to compounds reacting with the Folin-Ciocalteu reagent and FLAVA content refers to *p*-dimethylaminocinnamaldehyde (DMACA)-reactive substances. In the case of rooibos and honeybush the reaction is attributed to polymeric proanthocyanidin-type compounds, whereas monomeric flavanols are the major reactive polyphenols in green tea.

### 4.3. Cell Culture

Spontaneously immortalized keratinocytes (HaCaT) were a gift from the Department of Human Biology of the University of Cape Town (Cape Town, South Africa). Non-malignant normal fibroblast-like skin cells (CRL 7761) and basal carcinoma cell line (CRL 7762) skin cells obtained from the same patients were purchased from the American Tissue Cell Culture Collection (ATCC, Manassas, VA, USA). Cells were maintained in their respective media supplemented with 10% FBS and l-glutamine (2 mM) until confluency: HaCaT (RPMI-1640), normal cells (DMEM) and skin cancer cells (DMEM containing 0.12 mM HCl). Different passages of HaCaT (p70 to p80), normal (p16 to p20) and cancer (p15 to p22) cells were utilized in the experiments. 

### 4.4. Modulation of Cell Proliferation and Apoptosis

Cells were seeded in black solid (Porvair Sciences, Wraxhem, Wales, UK) and clear tissue culture 96-well microtiter plates for BrdU and caspase-3 assays, respectively, at a density of 5 × 10^3^ per well in their respective media (100 µL) containing 10% FBS. Cells were cultured for 24 h at 37 °C in 5% CO_2_/95% air to a confluency of 70% to 80%. Thereafter, the media were decanted and replaced with fresh media containing 0.5% FBS and the different dilutions of the various extracts. The plates were incubated for another 24 h and subjected to the different treatment protocols outlined below.

#### 4.4.1. Cell Proliferation Assay

Cell proliferation was determined using the BrdU chemiluminescent immunoassay kit following the manufacturer’s prescribed instructions. This assay quantifies cell proliferation based on the measurement of BrDU incorporation during DNA synthesis. Briefly, after 24 h incubation in the presence of the extracts the cells were labelled by adding the BrdU solution (10 µL) for 2 h at 37 °C. The media were then decanted and the cells fixed using a denaturing solution (200 µL) and an incubation period of 30 min at room temperature. After removal of the denaturing solution, cells were incubated in the presence of the BrdU antibody (100 µL) for 90 min. Plates were washed with saline (3 × 250 µL), treated with the substrate (100 µL), covered with foil and shaked for 3 min before quantification using a Veritas™ microplate luminometer (Promega, Madison, WI, USA). The luminescent signal was measured in relative light units (RLU) and the extent of cell proliferation expressed as a percentage (%) of the control treatment as follows:

% inhibition of cell proliferation = RLU_treated cells_/RLU_control cells_ × 100

IC_50_ values for % inhibition were calculated on the basis of the best fit for dose-response data using the 4-parameter logistic curve (sigmoidal variable slope) (GraphPad Prism version 5.04 for Windows, GraphPad Software, (La Jolla, CA, USA). The BrdU assay was conducted using four to five replicates of each dilution of an extract and the experiment was repeated at least twice.

#### 4.4.2. Cell Viability and Apoptosis Assays

The concentration range of the green tea and herbal tea extracts utilised for apoptosis encompassed the IC_50_ values generated in the cell viability and proliferation assays reported elsewhere [29,30]. The CellTiter-Glo^®^ Luminescent assay (Promega) was used for the determination of cell viability by monitoring the ATP content following the manufacturer’s instructions. IC_50_ values were calculated as described for cell proliferation. Apoptosis was determined for the cell lysates, following treatment with a cell lysis buffer (20 μL) in combination with one freeze-thaw cycle. Cell lysates were transferred (25 µL) into a white solid plate, the caspase 3/7 reagent (25 µL) added and incubated for 1 h in the dark at room temperature. The RLUs were determined using a Veritas™ microplate luminometer. Induction of apoptosis was calculated as a fold increase compared to the control, as well as expressed as a percentage of the viable cells. Staurosporine was used as the positive control and different concentrations were used for the premalignant (75 nM), normal (100 nM) and cancer (200 nM) cells. The negative control contained an equal volume of the buffer.

#### 4.4.3. Hoechst Stain

Normal cells were seeded (30 × 10^4^) in DMEM (1 mL) onto heat-sterilised cover-slips in small petri dishes (35 mm) and exposed to the different extracts at concentrations equaling their IC_50_ concentrations effecting reduction of cell viability and cell proliferation as obtained in the current study. Staurosporine (100 nM) was used as the positive control whilst cells exposed to an equal volume of buffer served as the negative control. The growth medium was discarded, the cells were washed with DPBS and incubated with 2 mL of 1 µg/mL Hoechst 33342 (prepared in medium) for 30 min at 37 °C, and then viewed under UV light using a Axoivert microscope (Zeiss, Göttingen, Germany) fitted with a blue filter (exclusion 358 nm, emission 461 nm). Magnification of 40× was used to record photographs.

### 4.5. Characterization of Pro-Apoptotic Activity of Rooibos by Flow Cytometry

The pro-apoptotic activity of the methanol extract of rooibos, at a concentration (0.32 mg/mL) was further characterised utilising normal cells in order to investigate the underlying cellular mechanisms of apoptosis. Cells were seeded in a 75 cm^2^ tissue culture flask (15 mL DMEM) at a density of 1 × 10^6^ and incubated for 24 h. The medium was discarded and cells incubated for 24 h in the presence of the methanol extract of rooibos. Cells were washed with HBSS, removed from the flask with a cell scraper and re-suspended in DPBS at a concentration of 1 × 10^6^/mL in 15 mL polystyrene sterile centrifuge tubes. Cells were then analysed by flow cytometry using the FACSCalibur™. (BD Biosciences, San Jose, CA, USA) for caspase-3 activity, membrane depolarisation (JC-1) and DNA damage.

#### 4.5.1. Caspase-3 Activity

The induction of caspase-3 activity was determined using the PE active caspase-3 apoptosis kit according to the manufacturer’s instructions. The assay utilises a rabbit caspase-3 antibody to detect the active form of caspase-3 in cells. Cells were washed twice with cold PBS (500 µL), pelleted by centrifugation for 5 min (300× *g*) and fixed using the BD cytofix/cytoperm solution™ (500 µL) for 20 min on ice. The supernatant was discarded, cells washed once with PBS (500 µL), re-suspended in BD Perm/Wash™ buffer (100 µL) plus antibody (20 µL) and incubated for 30 min at room temperature. After incubation cells were pelleted (300× *g*), washed with BD Perm/Wash™ buffer (1 mL) and re-suspended in BD Perm/Wash™ buffer (500 µL) for analysis by flow cytometry.

#### 4.5.2. Membrane Depolarisation

The effect of the rooibos methanol extract on mitochondrial integrity was assessed using the BD^TM^ mitoscreen (JC-1) kit following the manufacturer’s instructions. The kit utilises JC-1 (1st J aggregate forming cationic dye) which is sensitive to membrane potential changes. Incorporation of JC-1 monomers into the mitochondria is dependent on a polarised membrane potential and their accumulation results in the formation of concentration dependent red fluorescent aggregates. In depolarised membranes, JC-1 remains as monomers in the cytoplasm, exhibiting a lowered red fluorescence. Treated cells were re-suspended in DPBS (1 mL) and centrifuged at 400× *g* for 5 min at room temperature. The supernatant was discarded, the cells re-suspended in JC-1 working solution and incubated for 15 min at 37 °C in 5% CO_2_/95% air. Cells were pelleted by centrifugation (300× *g*), washed twice with assay buffer (1 mL), re-suspended in assay buffer (500 µL) and analysed by flow cytometry for JC-1 uptake in cells. 

#### 4.5.3. DNA Fragmentation

The ability of rooibos methanol extract to induce DNA damage during apoptosis was monitored, using the APO-Direct kit™ following the manufacturer’s instructions. The Apo-direct assay is a single step staining method that labels fragmented DNA to monitor apoptotic cells. Cells were re-suspended in PBS, centrifuged for 5 min at 300× *g* and after discarding the supernatant, fixed by re-suspending the pellet in 1% paraformaldehyde in PBS and incubated for 60 min on ice. The cells were again pelleted by centrifugation, the supernatant removed and washed twice with PBS and centrifuged. The pellet in the residual PBS was gently vortexed and the cells were stored overnight in 1 mL of 70% (*v*/*v*) ethanol at −20 °C. The ethanol was aspirated and the cells washed twice with wash buffer (1 mL) and centrifuged. The pellet was re-suspended in DNA labelling solution (50 µL) and incubated for 60 min at 37 °C, where after the cells were rinsed twice with the rinsing buffer (1 mL) and centrifuged. The pellet was re-suspended in PI/RNase solution and analysed for DNA fragmentation by flow cytometry.

### 4.6. Statistical Analysis

Significant group differences (independent classification variable) were evaluated using an ANOVA F1 test or the Welch’s test, depending if homogeneity of group variances were present when more than two groups were present by applying the generalised linear model procedure (SAS v. 9.4, SAS Institute Inc., Cary, NC, USA). Levene’s Test was used to test for the homogeneity of the variances and Tukey’s Test as the post-hoc test. When only two groups were compared the student t-test was used. When the original variables had non-parametric distributions, they were individually transformed to become parametric. The following data transformations were conducted for the methanol extracts: the square root for *C. sinensis*; the inverse transformation for *C. intermedia* and *C. subternata*. The Kruskal Wallis and Post hoc Tukey type tests were used to analyses data of the aqueous extracts of *C. sinensis* and *A. linearis* (nonparametric analyses). Statistical significance was measured at *p* < 0.05. Spearman’s rank correlation coefficients were determined to measure the strength of the relationship between the different variables.

## 5. Conclusions

Rooibos extracts and the aqueous extracts of honeybush alter cellular growth by a similar mechanism as green tea extracts, which involves the disruption of metabolic activity in the cell via mitochondrial membrane depolarisation. At a lower concentrations cell proliferation is inhibited whilst at a higher concentration apoptosis is induced, primarily targeting the removal of precancerous cells. The anti-proliferative activity of green tea and rooibos methanol extracts is mainly mediated by the monomeric type flavonoids which differ chemically. In contrast, the polymeric proanthocyanidin-like compounds, predominant present in the aqueous extracts of the honeybush species, is more likely to be involved in altering the cell growth parameters while the monomeric polyphenols tend to exhibit a protective effect. Specific polyphenol/cell interactions, which is extract specific, prevail in the chemopreventive properties of the herbal teas and this is reflected by polyphenol diversity that differs from green tea.

## Figures and Tables

**Table 1:** Anti-proliferative activity (BrdU IC_50_) of aqueous and methanol extracts of green tea and different herbal teas in skin cells.

Cell Type	Extract	Green Tea and Herbal Teas (mg/mL)			
		*C. sinensis*	*A. linearis*	*C. intermedia*	*C. subternata*
Premalignant cells	MeOH	0.035 ± 0.003 ^c^_A_ *	0.037 ± 0.005 ^c^_A_	0.350 ± 0.077 ^a^_A_	0.190 ± 0.019 ^b^_A_
	Aq	0.045 ± 0.007 ^c^_B_ *	0.068 ± 0.011 ^c^_B_	0.202 ± 0.037 ^a^_B_	0.164 ± 0.039 ^b^_A_
Normal cells	MeOH	**0.063 ± 0.009 ^c^_A_**	**0.058 ± 0.014 ^c^_A_**	0.151 ± 0.013 ^b^_A_	0.200±0.025 ^a^_A_
	Aq	**0.154 ± 0.021 ^b^_B_**	**0.208 ± 0.038 ^a^_B_**	**0.091 ± 0.022 ^c^_B_**	**0.098 ± 0.019 ^c^_B_**
Cancer cells	MeOH	0.035 ± 0.012 ^c^_A_	0.016 ± 0.003 ^d^_A_ *	0.150 ± 0.046 ^b^_A_	0.223 ± 0.030 ^a^_A_
	Aq	0.124 ± 0.025 ^a^_B_	0.048 ± 0.022 ^b^_B_	0.143 ± 0.037 ^a^_B_	0.158 ± 0.039 ^a^_B_

Values represent means ± standard deviations of five replication of at least two experiments. Means in a row (green tea and herbal tea groups) or column (extract type for a specific cell type) followed by the same letter (lower case superscript and upper case subscript, respectively) do not differ significantly, if letters differ then *p* < 0.05. Values in bold font for normal cells differ significantly from values of premalignant and cancer cells. Value in bold and italic font does not differ when compared to cancer cells. * Values differ significantly from normal and premalignant cells. Abbreviations: IC_50_—concentration yielding 50% inhibition of DNA synthesis; BrdU—5-bromo-2′-deoxyuridine; MeOH—methanol; Aq—aqueous; Premalignant cells—HaCaTs ; normal cells—CRL 7761; cancer cells—CRL 7762.

**Table 2:** Dose response effects of methanol and aqueous extracts of green tea and rooibos on the pro-apoptotic activity as a function of cell viability (ATP production) in skin cells.

Tea/Herbal Tea	Cell Type	Units	Methanol Extract			Aqueous Extract		
*C. sinensis*	Premalignant cells	**Extract (mg/mL)**	**0.080**	**0.050**	**0.035**	**0.170**	**0.114**	**0.038**
		Caspase-3_Fold	5.75 ± 1.26 ^a^	3.54 ± 1.00 ^a^	1.14 ± 0.22 ^c,^*	6.88 ± 1.65 ^a^	4.94 ± 2.43 ^a,b^	1.57 ± 0.76 ^c,^*
		% ATP production	36.12 ± 6.90 ^c^	49.08 ± 12.55^a^	79.19 ± 7.79^a^	47.49 ± 5.88 ^c^	67.63 ± 3.81^b^	83.67 ± 8.18 ^a^
	Normal cells	**Extract (mg/mL)**	**0.230**	**0.154**	**0.051**	**0.340**	**0.228**	**0.075**
		Caspase-3_Fold	4.33 ± 0.68 ^a^	1.98 ± 0.36 ^b^	1.22 ± 0.18 ^c,^*	5.58 ± 2.01 ^a^	3.20 ± 1.47 ^b^	1.07 ± 0.15 ^c,^*
		% ATP production	28.58 ± 4.22 ^c^	50.11 ± 1.50 ^b^	82.37 ± 5.04 ^a^	42.69 ± 14.23 ^c^	60.64 ± 15.10 ^b^	95.57 ± 26.22 ^a^
	Cancer cells	**Extract (mg/mL)**	**0.210**	**0.141**	**0.047**	**0.410**	**0.275**	**0.091**
		Caspase-3_Fold	2.13 ± 0.25 ^a^	1.79 ± 0.20 ^a^	1.26 ± 0.37 ^b,^*	1.55 ± 0.22 ^a^	1.35 ± 0.12 ^a^	1.13 ± 0.17 ^b,^*
		% ATP production	62.27 ± 8.70 ^c^	75.41 ± 7.10 ^b^	96.70 ± 8.59 ^a^	58.92 ± 4.58 ^c^	73.75 ± 6.09 ^b^	88.35 ± 7.24 ^a^
*A. linearis*	Premalignant cells	**Extract (mg/mL)**	**0.130**	**0.087**	**0.029**	**0.140**	**0.094**	**0.031**
		Caspase-3_Fold	4.67 ± 0.63 ^a,^	3.06 ± 0.28 ^a^	1.32 ± 0.40 ^b,^*	4.89 ± 0.78 ^a^	2.33 ± 0.59 ^b^	1.24 ± 0.25 ^c,^*
		% ATP production	41.74 ± 5.09 ^c^	68.12 ± 3.27 ^b^	83.04 ± 4.51 ^a^	50.41 ± 5.35 ^b^	69.76 ± 7.86 ^b^	76.36 ± 6.61 ^a^
	Normal cells	**Extract (mg/mL)**	**0.260**	**0.174**	**0.058**	**0.290**	**0.194**	**0.060**
		Caspase-3_Fold	2.91 ± 0.29 ^a^	2.46 ± 0.30 ^a^	1.57 ± 0.21 ^b,^*	1.80 ± 0.22 ^a^	1.71 ± 0.33 ^a^	1.58 ± 0.44 ^a,^*
		% ATP production	38.68±5.91 ^b^	54.29 ± 9.58 ^b^	89.76 ± 10.73 ^a^	41.09 ± 6.74 ^b^	54.11 ± 6.55 ^b^	78.65 ± 15.47 ^a^
	Cancer cells	**Extract (mg/mL)**	**0.310**	**0.163**	**0.016**	**0.260**	**0.154**	**0.048**
		Caspase-3_Fold	1.98 ± 0.17 ^a^	1.56 ± 0.19 ^b^	1.30 ± 0.24 ^b,^*	2.22 ± 0.25 ^a^	1.86 ± 0.26 ^a,b^	1.46 ± 0.39 ^b,^*
		% ATP production	54.53 ± 6.51 ^c^	72.00 ± 5.23 ^b^	90.60 ± 15.84 ^a^	51.72 ± 2.39 ^c^	63.19 ± 5.56 ^b^	87.05 ± 6.65 ^a^

Values represent means ± standard deviations of five replicates of at least two experiments. Statistically significant differences (*p* < 0.05) between green tea and rooibos concentrations (in a row) are indicated with different lower case letters in superscript. * Indicates no significant difference between extract concentration and control. Abbreviations: ATP—adenosine triphosphate; Premalignant cells—HaCaTs; normal cells—CRL 7761; cancer cells—CRL 7762. Negative control: caspase-3 fold increase (1.00 ± 0.14); % ATP production (100.00 ± 4.88).

**Table 3:** Dose response effects of methanol and aqueous extracts of *C. intermedia* and *C. subternata* (honeybush) on the pro-apoptotic activity as a function of cell viability (ATP production) in skin cells.

Herbal tea	Cell Type	Units	Methanol Extract			Aqueous Extract		
*C. intermedia*	Premalignant cells	**Extract (mg/mL)**	**0.720**	**0.360**	**0.180**	**0.520**	**0.260**	**0.130**
		Caspase-3 fold	1.12 ± 0.34 ^a,^*	1.34 ± 0.29 ^b,^*	1.17 ± 0.25 ^a,^*	3.40 ± 0.95 ^a^	1.81 ± 0.26 ^b^	1.32 ± 0.18 ^c,^*
		% ATP production	60.05 ± 12.31 ^b^	72.21 ± 11.72 ^b^	94.66 ± 4.73 ^a^	39.08 ± 2.50 ^c^	60.23 ± 8.09 ^b^	78.90 ± 10.09 ^a^
	Normal cells	**Extract (mg/mL)**	**1.370**	**0.760**	**0.150**	**0.500**	**0.295**	**0.091**
		Caspase-3 fold	1.09 ± 0.11 ^a,^*	1.10 ± 0.18 ^a,^*	0.98 ± 0.26 ^a,^*	1.86 ± 0.61 ^a^	1.42 ± 0.31 ^a,b^	1.16 ± 0.26 ^b^
		% ATP production	39.33 ± 4.46 ^c^	61.95 ± 11.40 ^b^	89.50 ± 20.60 ^a^	38.13 ± 12.42 ^b^	54.03 ± 11.80 ^b^	80.35 ± 20.17 ^a^
	Cancer cells	**Extract (mg/mL)**	**1.290**	**0.84**	**0.38**	**0.440**	**0.291**	**0.143**
		Caspase-3 fold	0.65 ± 0.17 ^c^	0.94 ± 0.14 ^b,^*	1.40 ± 0.23 ^a^	1.69 ± 0.32 ^a^	1.51 ± 0.13 ^a^	1.32 ± 0.20 ^b,^*
		% ATP production	49.57 ± 5.00 ^c^	66.6 ± 7.97 ^b^	95.48 ± 9.11 ^a^	48.65 ± 8.56 ^b^	63.32 ± 8.60 ^b^	92.17 ± 8.98 ^a^
*C. subternata*	Premalignant cells	**Extract (mg/mL)**	**0.468**	**0.234**	**0.117**	**0.417**	**0.209**	**0.104**
		Caspase-3 fold	1.73 ± 0.20 ^a^	1.46 ± 0.12 ^a^	1.10 ± 0.25 ^b,^*	3.27 ± 1.00 ^a^	2.03 ± 0.44 ^b^	1.45 ± 0.22 ^c,^*
		% ATP production	52.72 ± 10.24 ^c^	71.09 ± 7.65 ^b^	87.17 ± 9.40 ^a^	50.33 ± 7.21 ^b^	61.50 ± 8.53 ^b^	75.80 ± 6.51 ^a^
	Normal cells	**Extract (mg/mL)**	**1.080**	**0.640**	**0.200**	**0.370**	**0.230**	**0.098**
		Caspase-3 fold	1.25 ± 0.27 ^a,^*	1.39 ± 0.37 ^a,^*	1.14 ± 0.16 ^a,^*	2.11 ± 0.61 ^a^	1.77 ± 0.37 ^a,b^	1.45 ± 0.27 ^a,^*
		% ATP production	40.17 ± 6.89 ^c^	64.71 ± 5.99 ^b^	84.98 ± 8.28 ^a^	49.96 ± 6.09 ^c^	63.28 ± 7.45 ^b^	80.42 ± 9.88 ^a^
	Cancer cells	**Extract (mg/mL)**	**1.140**	**0.680**	**0.223**	**0.430**	**0.294**	**0.158**
		Caspase-3 fold increase	0.97 ± 0.26 ^a,^*	1.04 ± 0.12 ^a,^*	1.15 ± 0.06 ^a,^*	1.45 ± 0.33 ^a,^*	1.55 ± 0.35 ^a,^*	1.28 ± 0.23 ^a,^*
		% ATP production	36.09 ± 1.31 ^c^	60.68 ± 3.46 ^b^	76.79 ± 0.70 ^a^	39.68 ± 2.28 ^c^	66.38 ± 1.77 ^b^	82.00 ± 8.73 ^a^

Values represent means ± standard deviations of five replicates of at least two experiments. Statistically significant differences (*p* < 0.05) between honeybush extract concentrations (in a row) are indicated with differing lower case letters in superscript. Statistically significant differences (*p* < 0.05) between herbal tea concentrations (in a row) are indicated with differing lower case letters in superscript. * indicates no significant difference between extract concentration and control. Abbreviations: ATP—adenosine triphosphate; Premalignant cells—HaCaTs; normal cells—CRL 7761; cancer cells—CRL 7762. Negative control: caspase-3 fold increase (1.00 ± 0.14); % ATP production (100.00 ± 4.88).

**Table 4:** Correlation between caspase-3 activity and cell viability (ATP production) for cells treated with green tea and herbal tea extracts.

Tea/Herbal Tea	Methanol Extracts			Aqueous Extracts		
	Premalignant Cells	Normal Cells	Cancer Cells	Premalignant Cells	Normal Cells	Cancer Cells
*C. sinensis*	−0.881	−0.888	−0.800	−0.742	−0.878	−0.672
	(<0.0001)	(<0.0001)	(<0.0001)	(<0.0001)	(<0.0001)	(<0.0001)
*A. linearis*	−0.881	−0.802	−0.769	−0.745	−0.547	−0.724
	(<0.0001)	(<0.0001)	(<0.0001)	(=0.0001)	(=0.0002)	(<0.0001)
*C. subternata*	−0.841	−0.588	-	−0.783	−0.864	−0.554
	(<0.0001)	(=0.0004)	-	(<0.0001)	(<0.0001)	(=0.0027)
*C. intermedia*	−0.527	-	0.479	−0.819	−0.699	−0.599
	(=0.0005)	-	(=0.0015)	(<0.0001)	(<0.0001)	(<0.0001)

Spearman correlations were used to calculate correlation coefficients (R^2^) and *p* < 0.05 was considered statistically significant. Actual *p-*values are given in brackets. Premalignant cells—HaCaTs; normal cells—CRL 7761; cancer cells—CRL 7762.

**Table 5:** Concentration of TP, FLAVA and monomeric polyphenolic compounds of the methanol and aqueous extracts of green tea and herbal teas expressed as the TP/FLAVA ratios.

Tea/Herbal Extracts	Polyphenols *	Concentration * (µg/mg Extract)		TP/FLAVA Ratio	
		Methanol	Aqueous	Methanol	Aqueous
*C. sinensis*	TP	256.5 ± 36.9 ^A^	161.0 ± 20.8 ^B^	1.94	2.07
	FLAVA	132.3 ± 3.4 ^A^	77.6 ± 1.5 ^B^		
	EGCG	111.9 ± 3.0 ^A^	46.1 ± 1.5 ^B^		
	Total flavanols	200.8 ± 6.2 ^A^	100.0 ± 2.8 ^B^		
*A. linearis*	TP	350.7 ± 35.0 ^A^	250.5 ± 16.4 ^B^	12.92	13.92
	FLAVA	27.1 ± 0.8 ^A^	18.0 ± 0.6 ^B^		
	DHC	151.8 ± 1.7 ^A^	100.6 ± 1.8 ^B^		
	Total_ mono	209.1 ± 1.8 ^A^	138.1 ± 3.6 ^B^		
*C. intermedia*	TP	172.1 ± 4.1 ^A^	164.5 ± 11.3 ^B^	15.23	9.19
	FLAVA	11.3 ± 0.9 ^B^	17.9 ± 0.9 ^A^		
	Xanthones	87.8 ± 2.9 ^A^	54.0 ± 0.8 ^B^		
	Hesperidin	88.8 ± 11.6 ^A^	7.3 ± 0.6 ^B^		
	Total_ mono	186.2 ± 13.0 ^A^	64.0 ± 0.6 ^B^		
*C. subternata*	TP	220.5 ± 14.5 ^A^	175.0 ± 24.1 ^B^	16.96	7.64
	FLAVA	13.0 ± 0.6 ^B^	22.9 ± 0.9 ^A^		
	Xanthones	78.5 ± 1.3 ^A^	30.7 ± 4.4 ^B^		
	Hesperidin	63.1 ± 8.6 ^A^	8.0 ± 0.2 ^B^		
	Total_mono	174.4 ± 9.0 ^A^	51.3 ± 4.5 ^B^		

* Polyphenol levels of green tea and herbal tea extracts derived from Magcwebeba et al. [29]. Values represent means ± standard deviations. Significant differences (*p* < 0.05) in content values of the methanol and aqueous extracts (in a row) are indicated with different upper case letters in subscript. Abbreviations: TP—total polyphenol content; FLAVA—flavanol/proanthocyanidins; Total_mono—total monomeric polyphenols. *Camellia sinensis*—green tea: EGCG—epigallocatechin gallate; *Aspalathus linearis—*rooibos: DHC—dihydrochalcones (aspalathin and nothofagin); *Cyclopia* spp.—honeybush: xanthones (mangiferin and isomangiferin).
